# Contribution of K2P Potassium Channels to Cardiac Physiology and Pathophysiology

**DOI:** 10.3390/ijms22126635

**Published:** 2021-06-21

**Authors:** Salvador Herrera-Pérez, Ana Campos-Ríos, Lola Rueda-Ruzafa, José Antonio Lamas

**Affiliations:** Laboratory of Neuroscience, Department of Functional Biology and Health Sciences, CINBIO, University of Vigo, 36310 Vigo, Spain; camposriosana@gmail.com (A.C.-R.); lolarrzg@gmail.com (L.R.-R.)

**Keywords:** K2P channels, TWIK, TASK, TREK, heart

## Abstract

Years before the first two-pore domain potassium channel (K2P) was cloned, certain ion channels had already been demonstrated to be present in the heart with characteristics and properties usually attributed to the TREK channels (a subfamily of K2P channels). K2P channels were later detected in cardiac tissue by RT-PCR, although the distribution of the different K2P subfamilies in the heart seems to depend on the species analyzed. In order to collect relevant information in this regard, we focus here on the TWIK, TASK and TREK cardiac channels, their putative roles in cardiac physiology and their implication in coronary pathologies. Most of the RNA expression data and electrophysiological recordings available to date support the presence of these different K2P subfamilies in distinct cardiac cells. Likewise, we show how these channels may be involved in certain pathologies, such as atrial fibrillation, long QT syndrome and Brugada syndrome.

## 1. Introduction

Two-pore domain potassium (K2P) channels are the latest potassium channels family to be described. Since the discovery of the first member of the TWIK subfamily [1], the 15 known K2P channels cloned to date have been grouped into six different subfamilies: TIWK; TREK; TASK; THIK; TRESK; and TALK [2,3,4,5]. In mammals, K2P channels are expressed in both nervous and non-nervous tissue, and their distribution varies widely from the almost ubiquitous expression of TWIK to the weak pancreatic expression of TALK [6]. In the nervous system, K2P channels have been identified at both the central [1,7,8,9,10,11,12,13,14,15] and peripheral level, including the somatic [16,17,18,19,20] and autonomic nervous systems, where our group has contributed significantly [21,22,23,24].

**Figure 1:**
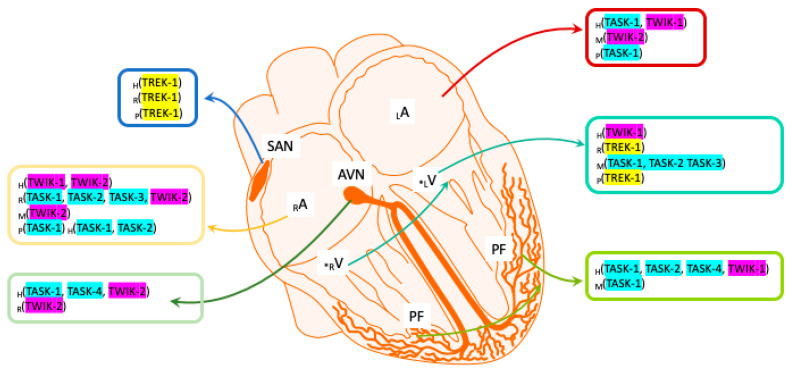
Scheme of the most prominently expressed K2P channels in the mammalian heart: TREK, TASK and TWIK. P, porcine; H, human; M, mouse; R, rat; SAN, sinoatrial node; AVN, atrioventricular node; PF, Purkinje fibers; RA and LA: right and left atrium; RV and LV: right and left ventricle—* right or left ventricular not specified.

Although the first K2P channel was not cloned until 1996, a barium-sensitive potassium current had already been identified in ventricular cardiomyocytes in 1993 using the Patch-clamp technique, and it had been proposed to be involved in the duration of the action potential (AP) plateau in guinea pigs [25]. Previously, a channel sensitive to changes in negative pressure, arachidonic acid (AA) and intracellular pH (pHi) had been demonstrated in rat cardiomyocytes [26], and even earlier, a potassium current sensitive to AA and mechanical stimuli had been identified in rat cardiomyocytes [27]. Significantly, the characteristics of these currents were consistent with the properties described later for some TREK channels of the K2P subfamily. Subsequently, several K2P channels were detected in cardiac tissue by quantitative real-time polymerase chain reaction (qRT-PCR), including TASK-1, TASK-3, TWIK-1, TREK-1, THIK, TALK-2 and TREK-2 [28,29,30,31,32,33]. However, in cardiomyocytes and nodal cells, the TASK, TWIK and TREK subfamilies were the most strongly expressed in mammals [34]. As such, we can now define where TASK, TWIK and TREK channels are expressed in the mammalian heart (Figure 1), although the expression of other K2P subfamilies in the heart seems to depend on the species analyzed. Here, we aim to bring together the most relevant information regarding cardiac TWIK, TASK and TREK channels, focusing on their putative roles in cardiac physiology and their involvement in coronary pathologies.

## 2. Two-Pore Domain in a Weak Inward Rectifying K+ Channel (TWIK)

As indicated above, TWIK-1 was the first human K2P channel cloned [1], and it is now considered to be part of the “tandem of pore domains in a weak inward rectifying K+ channel” (TWIK) subfamily that is made up of TWIK-1 (KCNK1), TWIK-2 (KCNK6) and TIWK-3 (KCNK7). TWIK channels are sensitive to changes in pHi, barium and quinine [35,36,37]. It was initially reported that TWIK-1 (also called cTBAK-1) is expressed more strongly in human ventricular myocytes than in atrial myocytes based on RT-PCR and Northern blot analysis [1,38]. However, TWIK-1 was later reported to be more intensely expressed in the atrium than in the ventricle [39,40,41], and it also seems to be expressed strongly in Purkinje fibers [42]. In murids, very strong expression of TWIK-1 was found in the whole rat heart, as confirmed by single-cell RT-PCR of both atrial and ventricular myocytes [29,43]. It was also shown that TWIK-2 is very abundant in the atrium and human atrioventricular node (AVN) [28]. Although this channel was thought to be expressed relatively homogeneously [37], it was later confirmed that TWIK-2 channels are more prominently expressed in the right atrium of both the human and rat heart [28], whereas no differences were observed in mouse ventricular or atrial cells [44].

Since TWIK mRNA is distributed widely in cardiac tissues, there is considerable information indicating that TWIK channels fulfil an important role in the physiology of the heart. In fact, it was proposed that TWIK channels might contribute to the heterogeneous cardiac inward rectifier potassium current named *I*_K1_ [38]. This current is mainly carried by Kir2.1 Kir2.2 channels [45,46,47], and it plays an important role in both the stabilization of the resting membrane potential (RMP) and in sculpturing the final phase of APs in muscle cells. Atrial fibrillation (AF) is one of the most widely studied heart disorders [48,49,50,51,52] and one of the most serious cardiovascular illness, clearly associated with the risk of heart failure [53]. The symptoms of AF include rapid and irregular palpitations, as well as fatigue and chest pain, with sinus irregularity usually underlying this disease. In humans, alterations to the expression of TWIK can contribute to the initiation or perpetuation of AF [40]. Specifically, reduced atrial TWIK-1 expression may be associated with chronic AF [39]. Alternatively, pathological, subphysiological K+ concentrations, known as hypokalemia, have also been related to AF, and TWIK-1 channels contribute to the stabilization of cardiomyocyte excitability under such conditions [54,55]. TWIK-2, another member of the TWIK subfamily, is very abundant in the atrium and human AVN, and since it is very sensitive to the antiarrhythmic drug dronedarone, it is thought to be a good target for the treatment of AF [28,56]. Finally, in patients with Brugada syndrome, a hereditary arrhythmic condition that causes sudden death, TWIK-1 is expressed strongly in Purkinje fibers [57], suggesting a putative role for TWIK channels is this pathological condition.

## 3. TWIK-Related Acid-Sensitive K+ Channels (TASK)

The TWIK-related acid-sensitive K+ channels (TASK) K2P subfamily is comprised of the TASK-1 (KCNK3), TASK-2 (KCNK5), TASK-3 (KCNK9), TASK-4 (KCN17) and TASK-5 (KCNK15) channels. TASK channels are extremely sensitive to pHe (extracellular pH) changes in the physiological range (6.0 to 7.8) and to limited O_2_ availability (hypoxia) [58,59,60,61]. Some members of the TASK subfamily are strongly expressed in rodent cardiac tissue relative to other organs such as the kidney or lungs, and TASK-1 is the K2P channel most strongly expressed in the rodent heart [58,62,63]. In cDNA libraries, moderate TASK expression was found in the mouse heart, yet in situ hybridization revealed strong TASK expression, especially in the atrium [58]. Broad expression of TASK mRNA was detected in the mouse heart in Northern blots [63], and strong right atrial expression was confirmed in the rat [28,29,62], with TASK subfamily members also expressed in the human heart [58].

Immunoblotting confirmed the presence of TASK-1 in other mammals such as pigs, where it is preferentially expressed in the atrium [64]. Moreover, TASK-1 mRNA was detected in the human right atrium, and moderate expression was detected in Purkinje fibers by RT-PCR [39,42,65], as well as in the AVN [34,66]. However, TASK-1 channels seem to have a preferential atrial expression in humans and rats, whereas both TASK-1 protein and RNA have been preferentially located in ventricular regions in mice [44,61]. This is also one of the most strongly expressed K2P channel in ventricular tissue, and in the bundle of His and Purkinje fibers in mice [67].

Like TASK-1, TASK-2 and TASK-3 are also weakly expressed in the rat heart when assessed by RT-PCR [28,29], and they are poorly expressed in mouse ventricular myocytes [30]. By contrast, TASK-2 channels are strongly expressed in the right atrium in humans, and they are moderately expressed by Purkinje fibers [42]. Likewise, strong TASK-4 expression has been detected by qRT-PCR in human Purkinje fibers and in the AVN [68]. Finally, TASK-5 was also detected in Northern blots of human heart tissue [59]. A specific TASK-1/TASK-3 blocker (the aromatic carbonamide, A293) produced a clear decrease in the macroscopic outward current in rat cardiomyocytes in response to steps from positive potentials to −40 mV, as detected in two-electrode voltage-clamp recordings [29]. The effect of A293 indicated functional expression of TASK-1 channels in heart tissue [29,30,44], and similar results were found in human right atrial cells in whole-cell patch clamp experiments [34].

The expression of mouse and human TASK-1 channels in heterologous system (COS, Xenopus oocytes) and their study using the Patch-clamp technique showed a clear TASK-1-like kinetic response to changes in pHe, with moderate sensitivity to Zn^2+^, quinidine and barium inhibition [43,61,62,66], in conjunction with insensitivity to changes in pHi and internal Ca^2+^ [58]. Both expression and functional data are clear indicators that TASK channels participate in cardiac physiology. Hence, TASK-1 channels could play an important role in shortening the length of APs, particularly as genetic (TASK^-/-^) or pharmacological ablation elongates AP duration (APD) in rodent [29] and human [30,34]. Furthermore, electrophysiological studies in Langendorff-perfused hearts showed that fibers from TASK-1^-/-^ mice had longer APs and a higher heart rate (HR) than TASK-1^+/+^ mice. In these conditions, no differences were seen in the P-wave and QRS duration, although there were clear differences in the QT interval that were enhanced in the knock-out (KO) animals [44]. Notwithstanding, a clear increase in both the QRS complex and APD was reported inTASK-1^-/-^ animals in vivo, yet not in the HR [30]. Since a substantial increase in the QT interval cannot be fully explained by changes in the APD in TASK-1^-/-^ mice, TASK-1 channels could influence the conductive capacity of heart tissues during development. Moreover, it has been speculated that the neuronal conduction system (composed of the His bundle, the branches and a conduction network of Purkinje fibers) in TASK-1^-/-^ animals could contribute to the lengthening of the QT interval [67,69]. In any case, it appears that TASK-1 channels can modulate the APD in human myocytes [66].

AF is the most common sustained arrhythmia [70], and patients with paroxysmal atrial fibrillation (pAF) and chronic atrial fibrillation (cAF) strongly overexpress the TASK-1 protein in the right atrium. In myocytes, TASK-1 channels contribute to AP repolarization and to background currents [64,66]. Since atrial myocyte AP shortening is the main mechanism underlying AF, it is reasonable to think that the overexpression of TASK channels in the atrium could trigger this cardiac alteration. The antiarrhythmic effect of TASK-1 is suppressed in pigs with AF [64], suggesting that these channels may constitute an important therapeutic target to stabilize the HR [39]. Conversely, it has also been assumed that TASK-1 downregulation is the basis of heart failure [70]. Therefore, it seems that as in other pathologies and for other K2P channels [71], the dysregulation (up or down) of TASK is correlated with AF.

Finally, since TASK-4 is strongly expressed in the conduction system of the heart, a human mutation that produces a clear gain in TASK-4 activity has been linked with severe conduction disorder, a hallmark of which is a progressive slowing of cardiac conduction [68,72].

## 4. TWIK-Related K+ Channels (TREK)

The TWIK-related K+ channel (TREK) subfamily includes the TREK-1 (KCNK2), TREK-2 (KCNK10) and TRAAK (KCNK4) channels [2,14,73]. TREK channels are especially sensitive to AA, changes in pHi and mechanical stimuli [15,74,75,76,77,78,79,80], as well as changes in temperature and membrane stretching [81,82]. In non-neural rodent cardiac preparations that mainly include cardiomyocytes and fibroblasts, the presence of TREK-1 channels has been largely confirmed by real-time RT-PCR and immunohistochemistry [28,33,74,83,84]. TREK-1 expression is quite heterogeneous, and in adult rats, there is less TREK-1 in epicardial than in endocardial myocytes [85,86]. In mice, TREK-1 channels are preferentially expressed in the ventricle [44], one of the most strongly expressed K2P channels in ventricular tissue [30]. However, in other animals such as pigs, a more uniform pattern of expression for TREK-1 is evident in atrial and ventricular myocytes [70]. The expression of TREK-1 channels in the adult human heart seems to be less important than in mouse [33], although there is intense TREK-1 mRNA in atrial appendages and the sinoatrial node (SAN) regions [34]. In fact, TREK-1 expression has been reported in SAN cells in the rat [28,83,86,87], mouse [88] and pig [70].

TREK-2 and TRAAK channels are considered to play a very limited role in the physiology of the heart [89,90]. Moderate TREK-2 and weak TRAAK expression has been reported in the rat heart by RT-PCR [28]. Unlike TREK-1 and TREK-2, the presence of TRAAK channels has not been confirmed in the mouse heart [29,91], although some splice variants of TRAAK channels have been located in human heart tissue [92]. As early as 1993, a possible role for a TREK-compatible activity in cardiac physiology was suggested [25]. Before TREK-1 was first cloned in 1996 [84], a new K+ conductance was found in neonatal rat atrial cells that responded to AA and changes in pHi. In inside-out patch-clamp experiments, the activity of these channels augments in the presence of AA and when the pHi is sequentially modified from 7.2 to 6.0 [27]. This activity is associated to TREK-1 or TREK-2 channels, because TRAAK channels are less sensitive to acidification than to alkalinization [14,15]. Furthermore, this channel showed a clear outward rectification at equimolar K+ concentrations, and the conductance of the individual channel measured during the outward current was twice that when measured during the inward current. With these data, we can speculate that the channels described in rat atrial cells corresponded to TREK-1-like channels. Finally, another TREK-like activity was identified when a stretch-activated K+ conductance was demonstrated in adult rat atrial cells [26], although a native TREK-1-like current was first characterized later in rat ventricular cardiomyocytes. In whole-cell experiments, an *I*_TREK_ current was activated by adenosine triphosphate (ATP) via AA release after phospholipase A_2_ (PLA_2_) activation [74]. The activation of this current produces a clear hyperpolarization [93] that could be attributed to TREK channels [94]. Finally, the functional presence of TREK-1 has been revealed in KO animals, both in cardiomyocytes [95] and pacemaker cells of the SAN, where it plays an essential role in repolarization and in spontaneous nodal activity [88].

The TREK subfamily also seems to play a relevant role in several pathological heart conditions. For instance, TREK-1 overexpression is evident in rat ventricular cardiomyocytes in situations of isoproterenol-induced hypertrophy [86], whereas experimental deletion of TREK-1 impeded the recovery of an ex vivo-induced ischemia phase. In vivo, these animals showed a significantly longer QT interval and higher postinfarction mortality [95]. Similar data were also obtained in the heart of TREK-1 KO mice, with a significantly longer R-R (time elapsed between two successive R-waves of the QRS signal on the electrocardiogram) and QTc intervals (the interval between depolarization and the corrected ventricular repolarization), suggesting clear SAN dysfunction with the possibility of bradycardia or supraventricular arrhythmias [88]. Porcine models of AF have shown a clear downregulation of TREK-1 in atrial regions [70], and it was suggested that TREK-1 fulfils a clear role in arrhythmiogenesis [88]. In this sense, dronedarone is one of the drugs most often used in this pathology and as other known antiarrhythmic drugs such as vernakalant clearly inhibit TREK channels [96]. These data support the involvement of TREK in fibrillary processes, and they make these channels potential therapeutic targets for the treatment of AF and other heart diseases [56]. Finally, diltiazem (a calcium channel blocker with antiarrhythmic effect) inhibits both TREK-1 and TREK-2 [97], reinforcing the hypothesis that TREKs, and more specifically TREK-1, play an essential role in cardiac pathophysiology [89,98].

From a functional point of view, TREK-1 appears to counteract the depolarizing effect produced by stretch-activated cationic currents, contributing to stimulation-activated feedback mechanics in the heart [83,99,100]. It has been suggested that its experimental withdrawal could be proarrhythmic [89]. In addition, the uneven expression of TREK-1 in different cardiac regions would contribute to a tighter control of the depolarizing wave generated during cardiac contraction [2]. In this regard, it is known that in epicardial myocytes of adult rats there is less TREK-1 than in endocardial cells [85].

## 5. Discussion and Conclusions

Although several K2P channels have been localized in cardiac tissue, the presence of TASK-1, TWIK-1 and TREK-1 seems to be particularly prominent [28,39,40,67,68,86]. K2P channels are expressed heterogeneously in the heart and in a species-dependent manner, and whereas TWIK-1 channels are more strongly expressed in the atrium than in the ventricles in humans, in murids this channel is expressed strongly throughout the heart [39,40,41]. Similarly, TASK channels are expressed specifically in the right atrium of the mouse and human heart but also in the AVN and Purkinje fibers [28,29,39,42,58,62,65]. Finally, TREK channels are preferentially expressed in the ventricles of mice [30,44], whereas they are more uniformly expressed in other animals such as pigs [70]. Finally, unlike TREK-1 and TREK-2, it is not clear that TRAAK channels are expressed in the heart of mice [29,91]. However, TREK-1 channels have been found in the atrial appendages and in the SAN of the human heart [33,34], and they are thought to be widely expressed in the ventricular tissue of mouse [30] and in both porcine nodes [70].

It is generally accepted that TWIK, TASK and TREK channels play an important role in stabilizing the RMP of several cardiac cells, including nodal and muscle cells [33,88,101,102]. In addition, TWIK, TASK and TREK channels contribute to AP repolarization, and they determine the APD [30,64,66,86]. In this regard, analyzing mRNA, protein and electrophysiological data suggest a clear role of K2P channels in cardiac physiology. Several K2P subfamilies, at least TASK, TWIK and TREK, have been associated with various pathological heart conditions [34,45,64,103,104,105,106,107]. Table 1 summarizes the current evidence regarding the relationship between these K2P channels and some of the most frequent heart pathologies. Interestingly, both TASK-1 upregulation and TWIK-1 downregulation in atrial cells could underlie the onset of AF or aid its perpetuation [39,64]. In this regard, it is generally accepted that TASK-1 is associated with AF [66,106]. By contrast, TASK-1 is expressed strongly in the conduction system [67], and therefore, it might represent a good therapeutic target for AF, at least as these patients show strong TASK-1 protein overexpression in the right atrium [34,65]. Interestingly, in models of AF, there is weaker TREK-1 expression in the right atrium but not in other cardiac regions [70]. Alternatively, when TREK channels are blocked, the most consistent change is a prolongation of the APD and TREK-1 channels that are overexpressed in the ventricular endocardium in experimental models of cardiac hypertrophy, a process linked with fatal ventricular arrhythmias [86]. Finally, the suppressive effect of antiarrhythmic drugs on TREK channels, such as dronedarone, diltiazem, verapamil (unpublished data) or vernakalant, strongly support the association of these channels with pathological conditions such as AF [91,96,97,106]. In general, both TREK-2 and TRAAK are considered to play a less important role than TREK-1 in heart physiology [89,90].

Brugada syndrome is an inherited disease characterized by an electrocardiographic abnormality and an increased risk of sudden cardiac death [108]. TWIK-1 is expressed strongly in this condition [57], suggesting a putative pathological role for these channels, while TASK-1 has also been implicated in this syndrome because it contributes to the lengthening of the QT interval [67,69].

Mechanical feedback is one of the most important subsystems that operates in the cardiovascular system [83,94,109], and in this regard, TREK channels are well-recognized stretch-activated K+ channels [33,77,78,110]. It is known that mechanical cardiac stimulation activates several ion currents, including nonspecific and K+ specific currents [85,111]. Hence, TREK channels could be good candidates to balance the depolarizing effect of the nonselective currents produced by the activation of TRP channels, contributing to the control of the mechano-electric feedback activated by mechanical stimulation of the heart [83,99,100].

It has become well established that TWIK, TASK and TREK are present in cardiac muscle and pacemaker cells. Parasympathetic neurons in the intracardiac ganglion (IG) are very relevant in the physiological control of the heart [112,113,114], and they are implicated in pathologies such as AF [51]. However, there are currently no data on the presence of K2P channels or their possible roles in these parasympathetic neurons. In light of the current data supporting the fact that K2P channels regulate the RMP in several types of neurons, it could be speculated that they also perform this function in the parasympathetic pathway, thus contributing decisively to its regulation. The control of cardiac function in resting conditions falls preferentially on the parasympathetic branch in the vast majority of mammals [115,116,117]; therefore, the possible presence of K2P channels in the parasympathetic pathway that innervates the heart should be analyzed in more depth. 

## Figures and Tables

**Table 1:** Established relationships between K2P channels and adverse cardiac conditions.

	Cardiac Condition	References
TREK	AF; CH; CF; L-QT	[56,70,86,87,88,95]
TASK	L-QT; AF; SB	[30,34,44,64,65,66,67]
TWIK	AF; BS	[40,42,54,57]

AF, atrial fibrillation; L-QT, long QT interval; BS, Brugada syndrome; CH, cardiac hypertrophy; CF, cardiac fibrosis.
